# Familial transmission of attention allocation towards one’s own and a peer’s body: An eye-tracking study with male adolescents and their fathers

**DOI:** 10.1371/journal.pone.0263223

**Published:** 2022-01-31

**Authors:** Rike Arkenau, Anika Bauer, Silvia Schneider, Silja Vocks

**Affiliations:** 1 Department of Clinical Psychology and Psychotherapy, Osnabrück University, Osnabrück, Germany; 2 Department of Clinical Child and Adolescent Psychology, Ruhr-University Bochum, Bochum, Germany; Union College, UNITED STATES

## Abstract

Previous research provides evidence of maternally transmitted body-related attentional biases in female adolescents. In contrast, it remains unclear whether a familial transmission of body-related attentional biases also exists within father-son dyads. Therefore, the current study examined *n* = 42 male adolescents and their fathers with respect to direct and indirect paternal influences on body-related attention patterns and specific body-related concerns in sons. Besides completing specific body image questionnaires, participants were shown pictures of their own and a respective peer’s body, while their eye movements were tracked. The fathers additionally viewed the body pictures of their own son and an adolescent peer. Contrary to the assumed direct and indirect paternal transmission processes, the sons’ body-related attention patterns were not significantly associated with the perceived amount of paternal body-related feedback, with the fathers’ attention patterns towards their own son’s and the adolescent peer’s body, or with the fathers’ attention patterns towards their own and the adult peer’s body. Similarly, no significant associations were found between direct or indirect paternal influences and the sons’ drives for muscularity and thinness, body dissatisfaction, and muscularity-related body-checking behavior. Comparing the present findings with previous research indicating a maternal transmission of body-related attentional biases and body-related concerns in female adolescents, alternative (not gender-linked) familial transmission processes, e.g., via one’s own mother, or a comparatively higher relevance of other sociocultural influences, e.g., via peers or the media, might be assumed for male adolescents.

## Introduction

Body dissatisfaction is defined as a general negative evaluation of one’s own body, including negative body-related thoughts and feelings [1]. Along with other facets such as frequent body checking or misperception of one’s own body dimensions, body dissatisfaction can be part of a disturbed body image [2, 3], which is included as a diagnostic criterion for anorexia and bulimia nervosa in the Diagnostic and Statistical Manual of Mental Disorders [4]. Body image disturbance also has been found to be associated with various other mental disorders, such as body dysmorphic disorder [5] and its subtype muscle dysmorphia [6].

Current concepts of body image disturbance in men not only include body weight or body fat dissatisfaction as significant aspects, but also incorporate muscularity dissatisfaction as well as distinct paths to disordered eating behaviors on the one hand, and muscularity-enhancing strategies on the other hand [7, 8]. According to previous research, body image disturbance, or facets thereof, might be widespread among the male population. For instance, Fallon et al. [9] found that about 38% of an adult male US-American sample reported being dissatisfied with their own weight, and about 22% stated that they were dissatisfied with their own muscle tone. Similarly, in a German sample, more than half of the examined male adolescents wished to have a more muscular body [10]. Previous studies have also consistently found that even at young age, male adolescents at least sometimes employ specific muscle-enhancing and/or weight-control behaviors, e.g., encompassing exercising, dieting, taking protein shakes and other supplements, or using anabolic steroids [11–13]. The clinical relevance of striving for a more muscular and lean body is highlighted by findings of a link to elevated levels of depression [14] and eating disorder pathology [15] and lower self-esteem [14, 16]. Moreover, various negative physical and psychological health consequences of using anabolic steroids have been documented, e.g., an increased risk of coronary artery disease, or clinically relevant mood changes (for a review, see [17]). This is especially relevant in view of previous findings indicating that the drive for muscularity is positively associated with the intention to use anabolic steroids [18]. Beyond that, a positive association between drive for muscularity and muscle dysmorphia symptomatology has been documented [19]. In sum, these findings highlight the need for a closer exploration of body dissatisfaction in men and its possible underlying mechanisms, e.g., in order to derive effective prevention or treatment strategies.

In terms of factors that might promote the development of muscularity- and weight-related dissatisfaction and associated body-modification strategies in men, current sociocultural models highlight the role of perceived pressures from the media, peers, partners, and parents. In this regard, the internalization of the lean-muscular male body ideal, and the tendency to engage in appearance-related social comparisons function as significant mediators (e.g., [7, 20–22]). Especially during adolescence, which marks a risk period for the development of eating disorder pathology [23], parents are assumed to represent important role models regarding their children’s body- and eating-related attitudes and behaviors [24]. Parents’ influences are considered to be direct or indirect in nature [24, 25]. Direct influences include appearance-related comments or gestures towards one’s own child and encouragement to use weight- or shape-control strategies [24, 25]. For instance, these may include parental comments on the child’s current weight or eating behavior (see [26]). By contrast, indirect influences, in line with a social learning process [27], comprise the child’s imitation of parental appearance-related attitudes or behaviors [24, 25]. More specifically, this means that the child notices a specific behavior displayed by the mother/father and then starts to show that behavior as well, e.g., begins to lift weights to modify his body, as he has observed his father doing (see [24, 28]). Although previous findings provide evidence for a gender-linked transmission model which assumes mothers to be more important for daughters and fathers for sons (e.g., [26]), fewer studies have examined father-son dyads. Corresponding to the findings reported for mother-daughter dyads (for a review, see [24]), these studies also indicate a positive association between direct influences (e.g., in the form of perceived negative paternal body-related comments or encouragement to lose weight) and the sons’ body dissatisfaction [26, 29], eating disorder pathology [29], and/or the desire for a thinner body [26, 30]. Furthermore, paternal comments perceived as referring to the relative importance assigned to physical appearance or other people’s weight or shape were found to be positively related to sons’ disordered eating symptoms [26, 29] and/or drive for thinness [26]. Similarly, perceived positive paternal body-related comments were related to a higher drive for thinness in sons [26], while no significant associations with sons’ body dissatisfaction levels or eating disorder pathology were found [26, 29]. In terms of indirect familial transmission, paternal modeling effects have been documented for sons’ engagement in muscle-enhancing behaviors [28], disordered eating symptoms, and specific dysfunctional body- and eating-related attitudes and behaviors, e.g., the frequency of weight loss attempts [31].

So far, these studies have focused primarily on self-report questionnaire data, while neglecting to examine associated cognitive processes, such as selective attention allocation towards eating- or body-related stimuli (for a review of current findings, see [32–34]). According to cognitive-behavioral models, these attentional biases are assumed to play a key role in the development and maintenance of eating disorder pathology or body image disturbance, e.g., by provoking negative body-related feelings, which in turn foster compensatory behavior, e.g., restrictive eating, to reduce the evoked negative affective states or the feared consequences [32, 33, 35]. Furthermore, as a form of social comparison, selective attention allocation towards one’s own and a peer’s body might also be relevant as a mediator of the association between perceived sociocultural influences and body dissatisfaction, or engagement in specific body-modification behaviors, respectively (e.g., [20, 21]). To explore selective body-related attention allocation and its association with various body-related concerns, previous studies primarily assessed participants’ gaze behavior towards body stimuli by using an eye-tracking paradigm [34]. These studies generally support the assumptions of the cognitive-behavioral models, for instance by finding evidence of biased attention allocation towards disliked areas of one’s own body in participants with body dissatisfaction or eating disorders (for a review, see [33, 34]).

To the best of our knowledge, the only study to have extended the research on selective body-related attention allocation to its potential familial transmission was conducted by Bauer et al. [36]. In non-clinical dyads of female adolescents and their mothers, the authors found evidence of both direct and indirect familial transmission [36]. More specifically, and indicative of direct familial transmission, it emerged that the more positively biased the daughter’s attention pattern towards her own compared to an adolescents peer’s body was (i.e., the more the daughter looked at her own attractive and the peer’s unattractive body parts in relation to the fixation times on her own unattractive and the peer’s attractive body areas), the less negative body-related feedback the daughter reported receiving from her own mother, and the more positively biased was the mother’s attention pattern towards her own daughter’s compared to the adolescent peer’s body [36]. Similarly, and indicative of indirect familial transmission, it was found that the more positively biased the daughter’s attention pattern towards her own compared to an adolescent peer’s body, the more positively biased was the mother’s attention pattern towards her own compared to an adult peer’s body [36].

As no previous study has explored the familial transmission of body-related attention allocation within father-son dyads, the present study aimed to transfer the approach described by Bauer et al. [36] to a non-clinical sample of male adolescents and their fathers. Based on the previous findings by Bauer et al. [36], we proposed the following hypotheses: First, the more positively biased the sons’ attention patterns towards their own compared to an adolescent peer’s body are (i.e., the more the sons look at their own attractive and the peer’s unattractive body areas as compared to their own unattractive and the peer’s attractive body areas), the less negative, and the more positive, body-related feedback they will report receiving from their fathers (hypotheses 1a and 1b, direct familial transmission). Second, the more positively biased the sons’ attention patterns towards their own compared to the adolescent peer’s body are, the more positively biased the fathers’ attention patterns towards their own son’s compared to the adolescent peer’s body will be (hypothesis 1c, direct familial transmission). Finally, we expected to find a positive association between the sons’ and the fathers’ attention patterns, each mirroring the gaze behavior towards subjectively attractive and unattractive body areas of their own and the respective peer’s body (hypothesis 1d, indirect familial transmission).

Furthermore, as only a small number of studies have examined direct and indirect familial transmission of body-related attitudes or behaviors within father-son dyads (for a review, see [24]), we sought to replicate previous findings, e.g., on paternally transmitted drive for thinness in sons (e.g., [26]), and to examine further variables which have so far received less or no consideration, such as drive for muscularity and muscularity-related body-checking behavior. Based on previous research [26, 28–31], we therefore proposed the following hypotheses: According to direct familial transmission, we expected perceived negative paternal body-related feedback to be positively associated with the sons’ self-reported drives for muscularity (hypothesis 2a) and thinness (hypothesis 2b), body dissatisfaction (hypothesis 2c), and muscularity-related body-checking behavior (hypothesis 2d). Due to fewer and less consistent previous findings, the respective associations with perceived positive paternal body-related feedback were examined on an exploratory basis. Furthermore, and in line with indirect familial transmission, we assumed positive associations between the sons’ and the fathers’ drives for muscularity (hypothesis 3a) and thinness (hypothesis 3b), body dissatisfaction (hypothesis 3c), and muscularity-related body-checking behavior (hypothesis 3d).

## Methods

### Participants and recruitment

A total of *n* = 42 male adolescents aged between 13 and 18 years (index group) and their fathers participated in the current study. Participants were recruited via press releases and email lists of Osnabrück University, Germany, notices in regional newspapers and on social media, and flyers at local gyms, sports clubs, and public buildings. Preliminary defined exclusion criteria for both the male adolescents and their fathers were acute suicidality and self-harm behavior. A present mental disorder was set as an additional exclusion criterion for the male adolescents, as representing the index group of the present study (see [36]).

### Measures and materials

#### Drive for Muscularity Scale

The drive for a more muscular body was assessed using the Drive for Muscularity Scale (DMS; [14], German-language version: [37]), which is a self-report measure encompassing 15 items rated on a 6-point Likert scale (1 = *always*, 6 = *never*). With the exception of item 10 (“I think about taking anabolic steroids.”), the items are categorized into an attitudinal (seven items) or a behavioral dimension (seven items). In the present study, the DMS total score was used only. As recommended by McCreary et al. [38], it was calculated excluding item 10. According to Waldorf et al. [37], the total score showed good internal consistencies in different samples of fitness-oriented or weight-training male adults (α = .89 - .90). In the present study, the Cronbach’s alpha was α = .91 for the sons and α = .90 for the fathers.

#### Eating Disorder Inventory-2

Participants’ drive for thinness and body dissatisfaction were assessed using the *Drive for Thinness* (DFT) and the *Body Dissatisfaction* (BD) subscales of the Eating Disorder Inventory-2 (EDI-2; [39], German-language version: [40]). The items of the DFT (seven items) and the BD (nine items) subscale are rated on a 6-point Likert scale (1 = *never*, 6 = *always*). In a non-clinical male adult sample, the internal consistencies of the DFT (α = .70) and the BD (α = .84) subscale were acceptable to good [40]. In the current study, the internal consistencies of the DFT and the BD subscale were α = .63 and α = .85 for the sons, and α = .74 and α = .75 for the fathers.

#### Male Body Checking Questionnaire

Participants’ muscularity-related body-checking behavior was assessed using the Male Body Checking Questionnaire (MBCQ; [41]). The self-report questionnaire consists of 19 items which are rated on a 5-point Likert scale (0 = *never*, 4 = *very often*). The MBCQ comprises four subscales (global checking, chest and shoulder checking, other-based checking, and body testing), although it is also possible to use the total score only [41]. The MBCQ showed acceptable to good internal consistencies (α = .72 - .86) in a male sample of undergraduate students [41]. For the purpose of the present study, the MBCQ was previously translated into German by a member of the larger work group. The internal consistency of the total score was α = .91 for the sons and α = .94 for the fathers.

#### Verbal Commentary on Physical Appearance Scale

Perceived negative and positive body-related feedback directed from fathers to sons was assessed using the *Negative Weight and Shape* (NWS; nine items), and the *Positive Weight and Shape* (PWS; five items) subscales of the Verbal Commentary on Physical Appearance Scale (VCOPAS; [42]), which was translated into German in a previous study [36]. Following Bauer et al. [36], the father was used as a specific source of feedback instead of the unspecific or unknown source used in the English-language original version. The items are rated on a 5-point Likert scale (0 = *never*, 4 = *very often*). Internal consistencies of the NWS (α = .84) and the PWS (α = .70) subscale were acceptable to good in a non-clinical adolescent female sample [36]. In the present study, Cronbach’s alpha was α = .77 for the NWS subscale and α = .75 for the PWS subscale.

#### Eye-tracking stimuli

Each participant was photographed from the neck down wearing identical neutral grey underpants and in four standardized positions (front view, back view, and both side views) by a male study assistant. The photos were taken with a Panasonic Lumix DMC-TZ8 digital camera, in front of a white screen, and under standardized lighting conditions. By using the four body pictures as well as the corresponding body pictures of a control male adolescent peer (age: 15 years, BMI: 19.74 kg/m^2^) or a control male adult peer (age: 40 years, BMI: 26.56 kg/m^2^), individualized photo presentations were created for each participant. The body pictures of the adolescent and the adult control peer were taken in advance under the same conditions.

#### Eye-tracking system

Spontaneous eye movements were recorded using the remote contact-free eye-tracking system *SMI RED 500* with an accuracy of 0.4°, a spatial resolution of 0.03°, a sampling rate of 500 Hz, and a distance of approximately 60 to 80 cm to the 22” computer monitor, on which the eye-tracking stimuli were presented (SensoMotoric Instruments, Teltow, Germany). Actual mean accuracy values, assessed directly before each eye-tracking session via a standardized 5-point calibration procedure, were *M* = 0.39 (*SD* = 0.11) for the male adolescents, *M* = 0.43 (*SD* = 0.18) for the fathers, and *M* = 0.42 (*SD* = 0.13) for the fathers’ second run including the body pictures of their own son and the adolescent peer. These results are in line with the recommendations of Holmqvist et al. [43] regarding adequate conditions for data recording in eye-tracking, and further correspond to those reported by Bauer et al. [36].

### Procedure

The study was conducted following the approach described by Bauer et al. [36]. Following an initial contact via telephone or email, and a preliminary agreement to participate in the study, father-son dyads were sent mail containing the written study information, the declaration of consent, also including a parental version for the adolescents aged < 18 years, as well as a questionnaire battery, e.g., on specific body-related attitudes or behaviors (see Measures and materials), which had to be completed at home before the eye-tracking assessment (processing time approximately half an hour). The written study information stated that the study aimed to assess body image in males, how male adolescents process their own and a peer’s body, and whether there are similarities between fathers and their sons. Furthermore, participants were informed about the photo shoot procedure, i.e., that for the purpose of the aforementioned study aims, they would be photographed in underwear and would look at the pictures of their own as well as the peer’s body. However, to prevent participants from intentionally influencing their gaze behavior, they were given a cover story that the study aimed to record pupil dilation, an autonomous process that cannot be affected intentionally.

The study took place at the laboratories of Osnabrück University, Germany, and lasted for about 1.5 hours per person. Although invited to attend together, fathers and sons underwent the study sections separately. Upon arrival, participants were asked to provide written informed consent and were screened with respect to acute suicidality and self-harm behavior by an M.Sc.-level clinical psychologist. Additionally, they were asked whether they suffer from a current mental disorder, e.g., a depressive or anxiety disorder. The screening results were validated by applying a structured clinical interview at the end of the assessment (see below). Subsequently, a male study assistant measured participants’ weight and height and took the body photos, and the individual photo presentations were then created. Whether participants first viewed their own or the peer’s body pictures was randomized by throwing a die (i.e., if the die landed on 1, 2, or 3, participants were assigned to the first group and if the die landed on 4, 5, or 6, they were assigned to the second group). The individual photo presentations were shown twice, with each body picture being displayed for 6 sec, subsequent to a centered fixation cross shown for 2 sec (also see [36]). Participants’ eye movements were recorded during the first trial. Therefore, participants were instructed to simply look at the body pictures. During the second trial, participants were then instructed to look closely at the body pictures in order to provide subsequent attractiveness evaluations by ranking 13 body parts (i.e., stomach, chest, shoulders, upper arms, forearms, thighs, lower legs, upper back, lower back, bottom, genital area, feet, and hands) of their own and the respective peer’s body pictures from most attractive to most unattractive. In an additional run, the fathers also viewed and assessed their own son’s and the peer’s body pictures, while undergoing the same procedure as described above. Finally, participants underwent a structured clinical interview conducted by an M.Sc.-level clinical psychologist. In line with the procedure described by Bauer et al. [36], the German version of the *Diagnostic Interview for Mental Disorders in Children and Adolescents* (Kinder-DIPS; [44]) was conducted with the male adolescents, and the *Diagnostic Interview for Mental Disorders* (DIPS; [45]) was conducted with the fathers. At the end of the experiment, participants were debriefed and rewarded with an expense allowance of 30 € each. The study design was reviewed and approved by the Ethics Committee of the Ruhr-University Bochum, Germany and was conducted in accordance with the Declaration of Helsinki.

### Data analysis

The eye-tracking data were processed and prepared for statistical analyses using the software BeGaze (SensoMotoric Instruments, Teltow, Germany). The analysis of the eye-tracking data was restricted to the frontal body pictures only, as this is common practice in previous research (e.g., [46]) and possibly provides the highest ecological validity, e.g., resembling the everyday perspective when looking in the mirror [36]. After examining the eye-tracking data quality by referring to the standards recommended by Holmqvist et al. [43] and the procedure described by Bauer et al. [36], the following areas of interest (AOIs) were defined for each of the frontal body pictures: shoulders, chest, upper arms, forearms, hands, stomach, genital area, thighs, lower legs, and feet. For this purpose, we used a standardized AOI template, which was initially created based on the adolescent and adult peer’s body and was then adapted to the individual characteristics of each participant. The individual AOIs did not overlap. To ensure accuracy, the AOI definition was conducted using standardized instructions and was checked for consistency by the first investigator (i.e., the first author). Subsequently, the variable *fixation duration* was extracted as the index of attention allocation towards a specific AOI, with a minimum fixation duration previously set to 100 msec for each fixation (see [36]). For each participant, we then summed up the extracted fixation duration for the three body areas rated as the most attractive and the three rated as the most unattractive, separately for one’s own and the peer’s body. Using these four newly created variables, we calculated two attentional bias scores which reflected the sons’ or the fathers’ gaze behavior towards their own in relation to the respective peer’s body as follows (see [36]): (attractive areas self + unattractive areas other)—(unattractive areas self + attractive areas other). Similarly, the fathers’ second attentional bias scores, which reflected the gaze behavior towards their own son’s in relation to the adolescent peer’s body, were calculated as follows: (attractive areas son + unattractive areas adolescent peer)—(unattractive areas son + attractive areas adolescent peer). By using this procedure, a positive bias score mirrors a positively biased gaze behavior and a negative bias score reflects a dysfunctional gaze behavior regarding one’s own body, or one’s own son’s body, respectively.

Statistical analyses were conducted using IBM SPSS 26. Direct and indirect familial transmission paths were examined using correlation analyses. In the case of normally distributed data (checked by visual inspection of histograms and by use of the Shapiro-Wilk test), Pearson’s product-moment correlation was calculated. Otherwise, Spearman’s rank correlation was used. With respect to the analyses involving the sons’ and/or the fathers’ bias scores reflecting the gaze behavior towards their own and the respective peer’s body, *n* = 9 father-son dyads had to be excluded due to inadequate eye-tracking data quality (see [36, 43]), resulting in a sample size of *n* = 33 father-son dyads for the analysis of hypotheses 1a, b, and d. For the same reason, one further father-son dyad had to be excluded from the analysis of hypothesis 1c, i.e., *n* = 10 father son-dyads were excluded, leaving *n* = 32 father-son dyads for the analysis of hypothesis 1c. For the analysis of hypotheses 2a-d and 3a-d, no participants were excluded in advance. However, as there were isolated missing data in particular questionnaires, in each case, the exact sample sizes are reported. The statistical significance level was set at *p* < .05, with Bonferroni-Holm corrections applied to account for multiple comparisons. The effect sizes of the correlation coefficients were quantified based on the recommendations by Cohen [47].

## Results

### Sample characteristics

Participants’ mean age and BMI, mean hours of physical training per week, and mean scores on the DMS, the DFT and the BD subscale of the EDI-2, as well as the MBCQ are displayed in Table 1. According to the Kinder-DIPS [44], none of the adolescents fulfilled the criteria for a mental disorder at the time of study participation. Among the fathers, the DIPS [45] indicated that *n* = 7 met the criteria for a current mental disorder (i.e., social anxiety disorder, recurrent depressive disorder, and insomnia) at the time of study participation. These participants were retained in the data set, as the exclusion criterion of a current mental disorder was set for the index group (i.e., the male adolescents) only. With respect to the educational level, the majority of the adolescents (*n* = 40, 95.2%) were school pupils (higher-track secondary school: *n* = 23, 54.8%; medium-track secondary school: *n* = 2, 4.8%; comprehensive school: *n* = 7, 16.7%; other school forms: *n* = 8, 19.0%). One adolescent (2.4%) reported that he was completing an apprenticeship, and one adolescent (2.4%) chose the category “other”. Among the fathers, *n* = 23 (54.8%) reported having a university degree or a university of applied sciences degree, *n* = 8 (19.1%) reported university entrance-level qualifications or an advanced technical college certificate, and *n* = 10 (23.8%) reported secondary school-leaving qualifications (missing: *n* = 1, 2.4%). In total, *n* = 33 sons (78.6%) lived together with both parents, *n* = 5 sons (11.9%) lived together with their mother only, and *n* = 2 (4.8%) lived together with their father only (missing: *n* = 2, 4.8%).

**Table 1:** Sample characteristics.

	Sons (*N* = 42)		Fathers (*N* = 42)	
	*n*	*M* (*SD*)	*n*	*M* (*SD*)
**Age**	42	14.88 (1.70)	41	49.54 (4.28)
**BMI**	42	20.63 (2.85)	42	26.97 (3.86)
**Physical training (hours/week)**	42	5.90 (2.78)	41	3.26 (2.31)
**DMS**	40	2.36 (0.92)	42	1.83 (0.72)
**EDI-2**				
Drive for Thinness	42	1.53 (0.57)	42	1.95 (0.71)
Body Dissatisfaction	40	2.16 (0.99)	41	2.56 (0.77)
**MBCQ**	41	0.77 (0.57)	42	0.44 (0.51)
**VCOPAS**				
Negative Weight and Shape	41	0.33 (0.42)	- ^a^	- ^a^
Positive Weight and Shape	40	1.13 (0.85)	- ^a^	- ^a^
**Fixation duration**				
Attractive areas own body	33	1232.76 (903.40)	33	640.94 (633.21)
Unattractive areas own body	33	1314.00 (993.42)	33	2108.61 (1173.79)
Attractive areas peer’s body	33	1188.94 (719.29)	33	770.85 (666.88)
Unattractive areas peer’s body	33	1187.39 (637.04)	33	1944.55 (956.21)
Attractive areas own son’s body	- ^b^	- ^b^	32	1176.56 (836.09)
Unattractive areas own son’s body	- ^b^	- ^b^	32	1586.94 (953.80)
Attractive areas peer’s body	- ^b^	- ^b^	32	1294.47 (692.60)
Unattractive areas peer’s body	- ^b^	- ^b^	32	1368.22 (809.03)

BMI = Body Mass Index; DMS = Drive for Muscularity Scale; EDI-2 = Eating Disorder Inventory-2; MBCQ = Male Body Checking Questionnaire; VCOPAS = Verbal Commentary on Physical Appearance Scale.

^a^VCOPAS scores were assessed for sons only.

^b^Eye-tracking data were assessed for sons only.

### Direct and indirect familial transmission

#### Body-related attention patterns

Contrary to hypotheses 1a and 1b (direct familial transmission), there were no significant associations between the sons’ attentional bias scores (*M* = -82.79, *SD* = 2129.35) and the sons’ mean scores on the NWS (*r*_s_ = -.242, *p* = .091, one-tailed, *n* = 32), or the PWS subscale of the VCOPAS (*r* = .287, *p* = .059, one-tailed, *n* = 31). Furthermore, and in contrast to hypothesis 1c (direct familial transmission), the sons’ attentional bias scores were not significantly positively associated with the fathers’ second attentional bias scores (*M* = -336.63, *SD* = 1683.94), reflecting the fathers’ gaze behavior towards their own son’s and the adolescent peer’s body (*r* = .143, *p* = .218, one-tailed, *n* = 32). Similarly, there was no significant positive correlation between the sons’ and the fathers’ first attentional bias scores (*M* = -293.97, *SD* = 1865.11), each mirroring the gaze behavior towards their own and the respective peer’s body (*r* = .197, *p* = .136, one-tailed, *n* = 33), thus contradicting hypothesis 1d on indirect familial transmission.

#### Body-related attitudes and behaviors

In contrast to hypotheses 2a-2d on direct familial transmission, when applying the Bonferroni-Holm corrected alpha levels to account for multiple testing, no significant positive associations emerged between the amount of perceived negative paternal body-related feedback and the sons’ mean scores on the DMS (*r*_*s*_ = .331, *p* = .020, one-tailed, *n* = 39), the DFT (*r*_*s*_ = .278, *p* = .039, one-tailed, *n* = 41) and the BD subscale of the EDI-2 (*r*_*s*_ = .335, *p* = .019, one-tailed, *n* = 39), and on the MBCQ (*r*_*s*_ = .233, *p* = .074, one-tailed, *n* = 40). Similarly, none of the associations between the perceived positive paternal body-related feedback and the sons’ mean DMS (*r*_*s*_ = .170, *p* = .307, *n* = 38), DFT (*r*_*s*_ = -.263, *p* = .101, *n* = 40), BD (*r*_*s*_ = -.383, *p* = .018, *n* = 38), and MBCQ scores (*r*_*s*_ = .234, *p* = .152, *n* = 39) were significant at the Bonferroni-Holm corrected alpha level. Furthermore, and contrary to hypotheses 3a-d on indirect familial transmission, no significant positive associations were found, i.e., between the sons’ and the fathers’ mean DMS scores (*r*_*s*_ = .176, *p* = .140, one-tailed, *n* = 40), mean DFT scores (*r*_*s*_ = .145, *p* = .179, one-tailed, *n* = 42), mean BD scores (*r*_*s*_ = .134, *p* = .208, one-tailed, *n* = 39), and mean MBCQ scores (*r*_*s*_ = .139, *p* = .193, one-tailed, *n* = 41).

## Discussion

According to previous research, there is evidence that body-related attentional biases are passed on from mothers to their adolescent daughters via direct and indirect familial transmission [36]. However, so far, there are no corresponding findings for father-son dyads. Therefore, the present study explored whether direct and indirect familial transmission of body-related attention patterns can also be found within dyads of male adolescents and their fathers. Additionally, the paternal transmission of specific body-related attitudes and behaviors was examined.

With respect to the examined familial transmission paths encompassing the sons’ and the fathers’ eye-tracking data, the findings were in contrast to the hypotheses. Specifically, the sons’ attentional bias scores, reflecting the gaze behavior towards their own and an adolescent peer’s body, were not significantly correlated with the perceived positive and negative paternal body-related feedback, thus contradicting hypotheses 1a and 1b on direct familial transmission. Similarly, in contrast to hypothesis 1c, which also assumed a direct familial transmission process, there was no significant positive association between the sons’ attentional bias scores and the fathers’ second attentional bias scores, reflecting how fathers inspected the body of their own son and the adolescent peer. Moreover, the assumed positive association between the sons’ and the fathers’ body-related attentional bias scores, each reflecting the gaze behavior towards their own and the respective peer’s body, was not significant, thus contradicting hypothesis 1d on indirect familial transmission.

Consequently, with the exception of the non-significant association between the sons’ attentional bias scores and the perceived positive paternal body-related feedback, the present findings are in contrast to those reported for a sample of female adolescents and their mothers, which indicated a direct and indirect maternal transmission of body-related attentional biases [36]. One possible explanation for these inconsistencies might be that body-related issues had not (yet) been relevant to the male adolescents in the present study, e.g., indicated by relatively low scores on the body image measures, potentially meaning that they were unaffected by direct or indirect paternal influences. Additionally, with a mean age of approximately 14 years, the male adolescents were approximately one year younger than the female adolescents examined in the study by Bauer et al. [36]. Considering gender differences in puberty-related physical changes, e.g., pubic hair and genital or breast development [48, 49], the male adolescents of the present study may have been at an earlier stage of pubertal development as compared to the aforementioned female adolescents in the study by Bauer et al. [36]. Moreover, as advanced puberty represents a potential risk factor regarding the development of body dissatisfaction and engagement in specific body-modification strategies (for a review, see [50]), a comparatively lower preoccupation with one’s own body might therefore be assumed for the male adolescents examined in the present study. Consequently, due to the specific study sample characteristics, potentially existing paternal transmission effects might not have been uncovered. Accordingly, we decided to conduct additional exploratory analyses to examine the potential influence of the sons’ age. When controlling for the sons’ age, partial correlations revealed no significant associations between the described variables. However, in line with our consideration, we found a significant positive association between the sons’ and the father’s viewing patterns towards their own and the respective peer’s body in 16-18-year-old male adolescents and their fathers (*r* = .667, *p* = .013, *n* = 13). In contrast, this association was non-significant within the younger subsample of 13-15-year-old male adolescents and their fathers (*r* = -.248, *p* = .292, *n* = 20). While these results might point to a paternal transmission of body-related attention patterns in older male adolescents, it should also be noted that the subsample sizes were small. Thus, future research should aim to replicate these findings in larger samples.

On the other hand, it could also be concluded that, contrary to prior expectations, fathers only play a subordinate role in transmitting body-related attention patterns to their adolescent sons. Instead, it may be assumed that male adolescents perceive stronger influences through their mothers, e.g., might be more likely to adopt the body-related attention pattern observed in their mothers, or might be more affected by maternal body-related feedback. To date, there are no studies exploring the relative influence of mothers and fathers with respect to body-related attentional biases in their children. However, previous research examining questionnaire data on parental influences (e.g., perceived negative body-related comments or dieting behavior) on specific dysfunctional body-related attitudes and behaviors in children indicated that mothers might generally be more influential for both girls and boys [29, 30]. As such, it is conceivable that mothers may also have a greater influence on their sons in terms of transmitting body-related attention patterns. Beyond this, it was shown that parental influences explained a higher amount of variance in body-related concerns in girls as compared to boys [26]. Accordingly, boys might be affected to a lesser degree than girls by their parents, and might instead be influenced more by other sociocultural influences, e.g., by (same-sex) siblings, peers, or the media (for instance, also see [21, 51]). With respect to the present findings, this means that male adolescents’ body-related attention patterns might have been affected to a higher degree by other sociocultural influences, which were not examined in the current study.

Finally, the discrepancy between the present findings and those reported by Bauer et al. [36] might also result from potentially influential mediators which were not considered. Specifically, past research with women and men has shown that the association between perceived sociocultural pressures to conform with a certain body ideal and specific dysfunctional body-related attitudes and behaviors is mediated by the internalization of the respective body ideal, and/or the tendency to engage in appearance-related social comparisons (see [20, 21, 52]). In line with this, previous research has indicated that in a sample of young men, the frequency of viewing “fitspiration” images on social media platforms was associated with lower body satisfaction through a more pronounced internalization of the muscular body ideal and a greater tendency to engage in appearance-related comparisons (e.g., [53]). In terms of the current findings, it might thus be assumed that these two variables also affect the degree to which a child’s body-related attention pattern is influenced by that of one’s parents, or by body-related parental feedback, respectively. Following this assumption, the lack of correspondence between the present results and those for mother-daughter dyads (see [36]) might be explained by potential gender differences in the internalization of the current sociocultural body ideal, and the tendency to engage in appearance-related social comparisons, with comparatively lower values among the male adolescents of the present study as compared to the female adolescents of the study by Bauer et al. [36]. This is supported by previous research showing that female adolescents/young women as compared to male adolescents/young men display a higher internalization of the media body ideal [54] and more frequently conduct body-related social comparisons [55, 56].

Besides exploring paternally transmitted body-related attention patterns in adolescent sons, the present study also examined direct and indirect paternal influences on sons’ body-related attitudes and behaviors. In contrast to the hypotheses on direct paternal transmission (hypotheses 2a-2d), there were no significant positive associations between the perceived negative paternal body-related feedback and the sons’ drives for muscularity and thinness, body dissatisfaction, and engagement in muscularity-related body-checking behavior after applying the Bonferroni-Holm-corrected alpha levels. To the best of our knowledge, there are no previous findings on paternally transmitted drive for muscularity and muscularity-related body-checking behaviors. However, the findings on drive for thinness and body dissatisfaction are contrary to findings reported for a male adult sample [29] and different male adolescent samples [26, 30]. These previous studies rather indicate that the more negative feedback the sons’ reported receiving from their fathers, the higher were their self-reported disordered eating symptoms and body dissatisfaction levels.

Focusing on the role of perceived positive paternal body-related feedback, no significant associations emerged with respect to the sons’ drives for muscularity and thinness, body dissatisfaction, and engagement in muscularity-related body-checking behavior. These findings are in line with a previous study in male adults, which also found no evidence of a significant association between perceived positive paternal body-related comments and the sons’ disordered eating symptoms and body dissatisfaction [29]. Again, studies exploring the association between positive paternal body-related feedback and the sons’ drive for muscularity and muscularity-related body-checking behavior are lacking. However, the lack of significant association between perceived positive paternal body-related feedback and sons’ drive for thinness in the present study is in contrast to the significant positive association reported by Rodgers et al. [26].

Regarding the examined indirect familial transmission paths (hypotheses 3a-3d), encompassing the sons’ and the fathers’ drives for muscularity and thinness, body dissatisfaction levels, and engagement in muscularity-related body-checking behavior, no significant associations were found. Hence, in contrast to previous research reporting evidence of a paternal modeling effect on sons’ engagement in muscle-enhancing behaviors [28], disordered eating symptoms, and specific dysfunctional body- or eating-related attitudes and behaviors [31], these present findings rather contradict the previously assumed indirect paternal transmission paths.

Possible reasons explaining the inconsistency between these present findings and those of the reported previous studies, indicating a direct and indirect paternal transmission of specific body-related attitudes and behaviors in male adolescents, might lie in differences in the specific study sample characteristics, particularly with respect to participants’ age. Taking a closer look, participants of the previous studies were mostly older than the male adolescents of the present study, i.e., were high school students with a mean age of approximately 16 years [26] or were already young adults, e.g., college students [28, 29, 31]. Hence, it might be conceivable that only when they become older (e.g., when transitioning from adolescence to young adulthood, or with an advanced pubertal status, respectively), are male adolescents’ body-related attitudes and behaviors affected by the perception or observation of direct or indirect paternal influences, e.g., as only then might body-related issues attain a higher subjective relevance. Beyond this, methodological differences with regard to the measures used to assess direct and indirect paternal influences on the sons’ body-related attitudes and behaviors might account for the described inconsistency between the present and previous findings (for a comparison, see [26, 28, 29, 31]).

Although this study extends previous research by being the first to explore whether body-related attention patterns are subject to direct and indirect paternal transmission, several limitations should be considered when interpreting or drawing conclusions from the presented results. The first limitation refers to the restricted representativeness of the recruited sample, particularly with respect to the age and the relatively high educational level of the sons and their fathers. Furthermore, the presented data were derived from non-clinical samples, hence precluding the generalization of the findings to a broader population, e.g., to male adolescents (or fathers) of other age groups, a different educational background, or to male adolescents (or fathers) with elevated levels of body dissatisfaction, or with related clinical diagnoses (e.g., eating disorders, or body dysmorphic disorder). Furthermore, it should be noted that there were five adolescent participants who primarily lived with their mothers. As this may potentially have diluted the hypothesized effects, future research should aim to assess further variables when including adolescent participants who live primarily with one parent, e.g., the age of the child at the time of parental separation or the exact amount of time spent with one’s father or mother. A further limitation refers to the fact that we did not conduct a priori power analyses. Considering the results of the post-hoc power analyses, with values below .80 for the obtained correlation coefficients, the current sample size may possibly have been too small to uncover the hypothesized effects. Consequently, future research should aim to replicate the present findings in larger and more diverse samples while using a prior power analyses to guarantee an adequate sample size. Another limitation might be seen in the relatively low internal consistency of the DFT subscale of the EDI-2 in the adolescent sample. Given that male adolescents are less likely to experience thinness-related body dissatisfaction than female adolescents and seem more likely to strive for a more muscular body (e.g., [57, 58]), it is questionable whether applying the DFT subscale provides reliable and valid results in male samples. Furthermore, the VCOPAS, which was used to assess paternal body-related feedback, was primarily developed in female samples (see [42]), and as such incorporates body-related feedback which might be less typical for men or male adolescents (e.g., as it focuses merely on weight- or thinness-related feedback). Consequently, possibly existing correlations, e.g., between paternal body-related feedback and the sons’ muscularity-related body-checking behavior, might not have been uncovered in the current study. Future research with males should thus aim to develop and apply measures which focus on muscularity-related body dissatisfaction or more clearly include muscularity-related feedback. Within this context, it might additionally be useful to assess whether items of already established measures function differently in females and males, e.g., to facilitate a gender-specific application of certain items or questionnaires (for instance, see [59]). Moreover, a further limitation refers to the fact that during the debriefing procedure, we did not ask participants about what they initially thought the main purpose of the current study was. Despite the applied cover story (i.e., that the study aimed to assess pupil dilation), it therefore remains unclear whether participants anticipated that their eye movements would also be tracked. As the awareness that their eye movements would be tracked could potentially have affected participants’ gaze behavior, future research should aim to include such questions during the debriefing procedure. Finally, it should be noted that the present data are correlational in nature. Hence, even though a paternal transmission effect (or the lack thereof) can be assumed based on previous theoretical frameworks [2] and empirical findings (e.g., [60]), future research projects should aim to take a closer look at the direction of the association, e.g., via experimental or longitudinal investigations.

As we examined a non-clinical sample of father-son dyads, future (eye-tracking) studies should also aim to examine whether the findings differ when including male adolescents or fathers with elevated levels of body dissatisfaction or specific associated clinical diagnoses (e.g., eating disorders or body dysmorphic disorder; for instance, see [61]). Due to the inconsistencies between the present results and those of previous studies, it would also be necessary to explore which factors could possibly have contributed to these deviations, e.g., the son’ age, or pubertal status. Finally, future research on male adolescents’ body-related attentional biases, attitudes, and behaviors should explore if there are direct and indirect influences of other potentially important social interaction partners, e.g., mothers, peers, or siblings, and should also take into account potentially mediating variables, e.g., the internalization of the current sociocultural body ideal or appearance-related social comparison tendencies (e.g., [20, 21]). Within this context, future research should also aim to preregister hypotheses and methodological procedures in order to enhance transparency and ensure the reproducibility of results.

## Conclusion

In summary, the present findings indicate that a familial transmission of body-related attentional biases, as it was found within female adolescents and their mothers [36], cannot be assumed for male adolescents and their fathers. Clinical implications derived for dyads of female adolescents and their mothers, primarily targeting the development of a positively biased body-related attention pattern among both daughters and mothers in order to prevent or reduce body image disturbance in female adolescents [36], thus seem to be less suitable for dyads of male adolescents and their fathers. Together with the non-significant findings concerning the hypotheses on direct and indirect paternal transmission of specific body-related attitudes and behaviors, the present findings lead to the conclusion that fathers might only play a minor role for their adolescent sons in terms of shaping body-related attention allocation, attitudes, and behaviors. However, considering that the power of the current study was possibly too low, future research should aim to replicate the present findings in larger and more diverse samples, while additionally exploring other potentially relevant sociocultural influences and mediators.
